# Carbohydrate Counting in Children and Adolescents with Type 1 Diabetes

**DOI:** 10.3390/nu10010109

**Published:** 2018-01-22

**Authors:** Giorgia Tascini, Maria Giulia Berioli, Laura Cerquiglini, Elisa Santi, Giulia Mancini, Francesco Rogari, Giada Toni, Susanna Esposito

**Affiliations:** Pediatric Clinic, Department of Surgical and Biomedical Sciences, Università degli Studi di Perugia, 06132 Perugia, Italy; giorgia.tascini@gmail.com (G.T.); mgiuliaberioli@gmail.com (M.G.B.); laura.cerquiglini@ospedale.perugia.it (L.C.); elisa.santi1988@gmail.com (E.S.); giu87manci@gmail.com (G.M.); rogari.francesco@virgilio.it (F.R.); toni.giada@gmail.com (G.T.)

**Keywords:** carbohydrate counting, glycosylated haemoglobin, hypoglycaemia, insulin, type 1 diabetes

## Abstract

Carbohydrate counting (CC) is a meal-planning tool for patients with type 1 diabetes (T1D) treated with a basal bolus insulin regimen by means of multiple daily injections or continuous subcutaneous insulin infusion. It is based on an awareness of foods that contain carbohydrates and their effect on blood glucose. The bolus insulin dose needed is obtained from the total amount of carbohydrates consumed at each meal and the insulin-to-carbohydrate ratio. Evidence suggests that CC may have positive effects on metabolic control and on reducing glycosylated haemoglobin concentration (HbA_1c_). Moreover, CC might reduce the frequency of hypoglycaemia. In addition, with CC the flexibility of meals and snacks allows children and teenagers to manage their T1D more effectively within their own lifestyles. CC and the bolus calculator can have possible beneficial effects in improving post-meal glucose, with a higher percentage of values within the target. Moreover, CC might be integrated with the counting of fat and protein to more accurately calculate the insulin bolus. In conclusion, in children and adolescents with T1D, CC may have a positive effect on metabolic control, might reduce hypoglycaemia events, improves quality of life, and seems to do so without influencing body mass index; however, more high-quality clinical trials are needed to confirm this positive impact.

## 1. Introduction

Type 1 diabetes (T1D), formerly called insulin-dependent diabetes or juvenile diabetes, is an autoimmune disorder characterised by a severe deficiency or absence of endogenous insulin, which results in chronic hyperglycaemia. The achievement of optimal glucose control is facilitated by intensive insulin treatment in patients with T1D [1,2]. The current recommendations are based on the basal bolus paradigm, with subcutaneous long-acting insulin or continuous subcutaneous infusion of rapid-acting insulin to cover basal requirements and with rapid-acting insulin to prevent or correct glucose excursions. Frequent self-measurements of capillary blood glucose allow for insulin dose adjustments, with the aim of achieving good metabolic control [3]. Furthermore, continuous glucose monitoring (CGM) provides information about the glucose level every 5 min and allows patients to view their glycaemic data either on their insulin pump or on a separate receiver [4].

Despite advances in medical treatment and technology, nutritional therapy continues to be a cornerstone of diabetes care [5]. Nutritional recommendations for a healthy lifestyle for the general population are also appropriate for children and adolescents with T1D, with the only difference compared to healthy peers being the need for insulin therapy. The International Society for Paediatric and Adolescent Diabetes (ISPAD) Consensus Guidelines of 2014 stressed the importance of and special issues applying to nutritional management for children with diabetes [6]. The Guidelines recommended that a proper diet should allow for optimal growth, maintain an ideal weight, and prevent acute and chronic complications. The approximate energy intake and essential nutrients should be distributed as follows: carbohydrates 50–55%; fat 30–35%; and protein 10–15%.

Carbohydrates are the primary macronutrient that has an effect on the postprandial glycaemic response; their dietary intake should not be limited to ensure proper growth in children and adolescents with T1D. Adjusting the insulin dose to carbohydrate intake could produce potential improvements in glycaemic control and quality of life. Carbohydrate counting (CC) is a meal-planning tool for patients with T1D, based on an awareness of foods that contain carbohydrates and their effect on blood glucose. The bolus insulin dose needed is obtained from the total amount of carbohydrates consumed at each meal and the insulin-to-carbohydrate ratio (ICR) [7]. The current guidelines recommend the algorithms for prandial insulin be based on the carbohydrate amount taken in during a meal. They recognise the additional benefits to glycaemic control due to the use of the glycaemic index (GI) and that fats and proteins in the diet may influence postprandial glycaemia [6,8,9]. The aim of this review is to analyse the impact of CC in children and adolescents with T1D.

## 2. Educational Program on Carbohydrate Counting (CC)

With the discovery of insulin, researchers recognised that the total amount of dietary carbohydrates should be used to determine the need for insulin at meals. In the 1980s, the conventional insulin therapy imposed very rigid eating patterns with restricted and controlled carbohydrates portions. Subsequently, in the 1990s, the Diabetes Control and Complication Trial (DCTT) was a turning point for the treatment of diabetes. From this study, it was found that intensive insulin therapy, using multiple daily injection (MDI) or insulin pumps (CSII), improved glycaemic control and reduced complications related to the disease. In the DCTT, CC, one of the methods of meal planning, was considered effective in achieving good glycaemic control and allowing more flexibility in food choices [10,11].

An experienced team, including the role of the dietitian, is essential for the patient with T1D and his or her family so that they can then partake in the educational program and learn the method of CC as well as healthy-eating principles [11]. Children and those who care for them can learn to estimate the amount of carbohydrates with reasonable accuracy; however, continuing education therapy is needed. Carbohydrates have the most significant impact on raising postprandial blood glucose. A careful counting of carbohydrates will lead to the correct calculation of the required insulin dose, which in turn will lead to normalising postprandial glycaemia [12]. The American Academy of Nutrition and Dietetics have identified three levels of CC [8]:

- Level 1, Patients should understand that carbohydrates raise blood glucose, and they must be encouraged to consume a consistent amount of carbohydrates per meal. Simple methods to quantify the carbohydrates must be provided: gram increments of carbohydrates, 10–12 g carbohydrate portions, and 15 g carbohydrate exchanges. In particular, this level is helpful for patients in therapy with twice-daily insulin doses and who need consistent carbohydrate intake [13];
- Level 2, Pattern management. This represents an intermediate step, in which patients learn to evaluate changes in blood glucose compared to carbohydrates consumed, which are modified by insulin and physical activity. Therefore, patients supported by paediatric teams can make changes to their insulin. However, ever more frequently, paediatric teams use other methods, such as carbohydrate intake or the insulin-to-carb ratio (ICR);
- Level 3, Advanced CC. Patients in MDI or CSII learn ICR and how to use it.

## 3. Insulin-to-Carb Ratio (ICR), Insulin Sensitivity Factor (ISF), and Glycaemic Index (GI)

The insulin-to-carb ratio (ICR) is individualised and depends on one’s sensitivity to insulin, which is how many grams of carbohydrates 1 unit of insulin covers. ICR allows children to obtain their insulin needs at mealtimes on the basis of the carbohydrates that will be consumed at that time, their blood glucose level, and their anticipated physical activity [6,9]. For the identification of the carbohydrates-to-insulin ratio, the empirical method of the 500 rule or 300–450 rule, for very young children who need less than 10 units of insulin a day, is often used [14,15,16,17]. It consists of dividing 500, 300, or 450 by the total daily insulin dose (TDD). ICR is not constant during the day; it tends to be higher in the morning, lower for lunch, and higher in the evening [18]. ICR varies more in children than in adults, which is linked to the more frequent changes occurring in the daily activity of the child [9].

The insulin sensitivity factor (ISF) is a correction algorithm of pre-prandial glycaemia. In particular, ISF indicates how many mmol/L (or mg/dL) 1 unit of insulin lowers blood glucose by; it is obtained by dividing 1800 (rapid analogue) or 1500 (regular insulin) by the TDD [19]. ISF also needs to be individualised for each patient. Regarding infants and toddlers, ISF is generally higher, approximately 100–150 mg/dL [19]. A recent study showed that the bolus insulin for meals was more than the one calculated by the 500 rule, in particular at breakfast and in pre-pubescent children. Moreover, for corrections, insulin need was slightly less than that calculated by the 100 rule (100 divided by total daily insulin dose) [19].

The GI expresses the glycaemic response after eating a known amount of carbohydrates contained in food, but in relation to the same amount of carbohydrates contained in white bread. The glycaemic area measured 120 min after food intake is expressed as a percentage of the standard. Using the GI and eating low-GI food may produce modest benefits in the control of postprandial hyperglycaemia. Moreover, to consider both the quality and the amount of carbohydrates, the concept of glycaemic load was introduced [20]. In patients with T1D, GI should be used with a method of carbohydrate quantification [21].

### 3.1. Glycaemic Control

In previous studies conducted in adult subjects, glycaemic variations were assessed after the ingestion of different amounts and qualities of carbohydrates: complex carbohydrates in a mixed meal and simple sugar dextrose [22]. Blood sugar was higher, but for a shorter time, after the dextrose load than after the mixed meals. The total amount released by the artificial pancreas to return glycaemia to basal levels did not differ when exposed to different types of carbohydrates, but a difference did occur in the release kinetics. Furthermore, there was a linear relation between carbohydrate intake and insulin need.

A systematic review and meta-analysis was recently performed to evaluate the effectiveness of CC on glycaemic control in people with T1D [22]. It included 10 studies, published from 2000 to 2014, involving 773 participants. Four studies enrolled children and adolescents [23,24,25,26], and six studies included adults [27,28,29,30,31,32]. Overall, CC, compared with other diabetes diet methods or usual diabetes diets, improved HbA_1c_ values, in line with a previous systematic review and a meta-analysis [28,33,34]. A non-significant improvement of HbA_1c_ was found in the subgroup analysis restricted to trials that compared CC with other T1D diet methods [14].

One systematic review and meta-analysis aimed to summarise all available evidence from both observational and randomised controlled studies on the effects of advanced CC on glycaemic control, psychosocial measures, body weight, and severe hypoglycaemic events in patients of all age groups with T1D [26]. Six randomised controlled trials and 21 before/after studies were included; four uncontrolled trials (Table 1) [35,36,37,38] and one randomised controlled trial (Table 2) [39] were conducted in children and adolescents with a mean age ± standard deviation of 12 ± 4 years. In only one study, in which 28 adolescents were included, the improvement in HbA_1c_ was not demonstrated after advanced CC. These 28 patients switched from a prescriptive to a flexible meal plan and insulin. According to the authors, this result could be related to inaccurate CC, incorrect calculation of insulin dose, or inappropriate insulin injection technique.

Bell et al. only included one study of children in their systematic review [28]. The results favoured the alternative approach, i.e., a low glycaemic index over CC. However, the improvement of the glycaemic control found in adults needed to be interpreted with care, as more studies had a concurrent intensification of insulin therapy in the intervention group.

Compliance and the ability to accurately estimate the amount of carbohydrates consumed by children and adolescents with T1D is another aspect that should be considered. The greater ability of parents to count carbohydrates led to lower values of HbA_1c_ in their children and adolescents [40,41,42,43,44]. At the same time, there are wide variations in this ability, with studies where participants may estimate carbohydrates to within 10–15 g or within 15–20% of the actual amount, while in others, only half could accurately evaluate the carbohydrate amount or had large differences in calculations [28,40,41,42,43,44,45].

An important aspect that should not be overlooked is the effect of time on changes in HbA_1c_. The effects of diabetes self-management training programmes were short-lived. Thus, as recommended in many countries, regular reinforcement of advanced CC is critical in patient management [41,46,47]. In the review by Schmidt et al., the study with the longest period of evaluation was also the study with the smallest improvement in HbA_1c_ [34]. The accuracy of counting carbohydrates is associated with an improvement in glycaemic control; at the same time, intense glucose monitoring is an independent variable related to the reduction in HbA_1c_ [43,48].

Considering the available data, in several studies the CC method has been compared to a control arm consisting of usual or standard care rather than alternative methods for calculating bolus insulin. In addition, observational studies generally conclude showing no improvement in glycaemic control. Furthermore, results in the paediatric and adolescent population seem better than those observed in adults, probably also for the psychological aspects associated with CC. All these considerations highlight the need of further well-designed, double-blind large studies that confirm the role of CC in patients with T1D of different age groups.

### 3.2. Hypoglycaemia

The calculation of carbohydrate amount for the adjustment of insulin dose is useful for better glycaemic control, and, for the practical CC, the small quantification errors are permissive. A 10 g variation in the carbohydrate quantity did not induce differences in blood glucose. Furthermore, patients may have episodes of hypo- and hyperglycaemia for an overestimation or underestimation of 20 g in a meal of 60 g carbohydrates [35,37,49]. Adolescents may be less accurate in the calculation and have a decay of glycaemic control. Hypoglycaemia is not reported or defined uniformly [40]. Uncontrolled studies showed a significant reduction in severe hypoglycaemia in children with T1D using CC [29,30]. Other data suggested that CC did not significantly decrease hypoglycaemia events [14]. In a before/after study in nine children, an insignificant increase in non-severe hypoglycaemia was observed [28]. CC might reduce the frequency of hypoglycaemia; probably, the reduction in HbA_1c_ concentration is a result of stabilised glycaemic control rather than just an overall lowering of blood glucose [15].

### 3.3. Growth, Weight, and Psychological Effects

Intensive and appropriate insulin therapy and, consequently, better metabolic control have enabled an improvement in the growth prognosis of paediatric patients with T1D [30,50]. Modern T1D care prevents abnormalities of the GH-IGF-I axis, which means that children with T1D can achieve a final height similar to their healthy peers [51].

Some clinicians believe that flexibility in the diet, which can be permitted by CC, may lead to the development of obesity [8,9]. The DAFNE study demonstrated that a freedom diet did not increase cardiovascular risk factors, including obesity [26]. Paediatric studies have shown that therapy MDI associated with the calculation of carbohydrates does not cause obesity in children with T1D. Some studies documented a decrease in body mass index (BMI), and some studies reported an increase; nevertheless, only minor variations in weight after the use of CC were found [30,52,53,54]. The increased weight documented in children and adolescents with T1D was attributed to changes in growth occurring during puberty progression [29]. In another study, Marigliano et al. evaluated the impact of CC and nutritional education on changes in dietary habits, body fat composition, and fat body distribution in diabetic children in therapy with CSII [55]. The results revealed that CC and a proper nutrition education did not have a negative effect on dietary habits, body composition, or body fat distribution. Moreover, a reduction of intake of fat and protein, with an increase in carbohydrates intake, was observed in a subgroup of subjects showing a significant improvement in metabolic control.

Females at a young age with T1D face a higher risk of eating behaviour disorders and, consequently, are more likely to have impaired metabolic control and diabetic complications [56]. Furthermore, a cross-sectional case-controlled study has shown that there was an increased frequency of food disturbances in pre-teen and early-teenage girls with T1D [57]. Rigid dietary patterns that do not conform to the habits and needs of patients might have an effect on the psychological development of children with T1D. An Australian 9-month before/after study that evaluated diabetes-related quality of life reported significant improvements in patient-rated life satisfaction after the introduction of CC. Often, the lack of flexibility in the management of meals and snacks is one of the worst aspects of living with diabetes, and adolescents especially ignore dietary advice. Thus, CC is an important tool allowing children and teenagers to manage their T1D more effectively within their own lifestyles [1,31]. This psychological effect of CC could also be useful during the honeymoon period and the transition from adolescence to adulthood.

In addition, it is extremely important to highlight that for the children and adolescents with T1D, the psychological aspects, such as the quality of life, adherence, and motivation, are key points of good glycaemic control. The reports of the Dose Adjustment for Normal Eating (DAFNE) study emphasised the positive effect of CC on the quality of life [58]. So far, many kinds of structural education programs for T1D have been reported [59,60]. Most of these programs included CC and showed the improvement of patients’ adherence, as well as motivation. Therefore, CC has a positive effect on patients’ adherence and motivation too. In addition, it has been observed that CC also has a positive effect on the parents and caregivers, who are the key persons for the treatment of infants and young children with T1D [59,60].

### 3.4. Bolus Calculator

The bolus calculator is a useful tool both for patients with T1D who require MDI and patients with an insulin pump, which allows the calculation of the prandial insulin dose based on the amount of carbohydrates, the pre-prandial blood glucose value, and active insulin [61]. Its use is associated with improved metabolic control and greater satisfaction in treating adults, children, and adolescents [62]. Few studies found the bolus calculator to be effective in children using CSII. The bolus calculator and CC may have beneficial effects in reducing HbA_1c_ by improving post-meal glucose, with a higher percentage of values within the target range [63,64,65]. Moreover, an insulin pump offers the possibility to tailor insulin delivery to the meal composition through the use of different bolus options: normal, dual wave, or square wave. Evidence suggests that children who apply at least one D-W/S-W bolus a day are better at achieving the recommended HbA_1c_ level [66]. In an 18-month observational study, Rabbone et al. investigated the effect of CC, with or without an automated bolus calculator, in children treated with MDI. This study showed that bolus calculator use led to greater improvements in HbA_1c_ [67].

### 3.5. Fat and Protein Counting

Learning about CC is important because it also increases knowledge about other macronutrients [57]. Low carbohydrate diets have been popularised by social media for those who wish to lose weight [68]. However, the promotion of a low carbohydrate diet in lay media is in contrast to published paediatric diabetes guidelines that endorse a balanced diet from a variety of foods for optimal growth and development in children with T1D [69]. De Bock et al. described a series of six cases where the adoption of a low carbohydrate diet in children impacted growth and cardiovascular risk factors with potential long-term sequelae [69]. Meals enriched in fat and protein result in a prolonged blood glucose rise by 3–4 h after food ingestion, and frequently, a relative insulin resistance is observed [70]. The American Diabetes Association recommends an appropriate glycaemic impact education regarding protein and fat. Recent studies and the use of continuous glucose monitoring have shown that post-prandial glucose excursion may occur because of fat, protein, and GI [71,72]. CGM and increasing focus on the daily lives of patients highlight the limitations of the traditional CC method, used to calculate the meal-time insulin dose. From the new insights about the effect of dietary macronutrients on post-prandial glucose control, it appears that, to improve the calculation of the insulin bolus, CC should be integrated with the counts of fats and proteins. On the other hand, it has been demonstrated that bolus calculation for high fat feeding prevents late rises in postprandial triglycerides and tumour necrosis factor alpha, thus improving cardiovascular risk profile [73]. Therefore, the development of suitable and usable algorithms is necessary, without forgetting the importance of educational therapy for a successful translation of fat/protein counting in real life [74].

## 4. Conclusions

Current guidelines recommend that the algorithms for prandial insulin dose be based on the carbohydrate amount taken with a meal. CC and intensive insulin therapy enable patients to have greater freedom in the management of the meals or snacks and activities of daily living, without forgetting the principles of healthy eating. A paediatric interdisciplinary T1D care team is fundamental. It has to provide education, monitoring, and support to the children and their caregivers, bearing in mind crucial aspects of children and adolescents with T1D: physical and psychological development and T1D complications.

CC may improve metabolic control and reduce HbA_1c_ concentration. Moreover, with developments in medical technology and with the new insights into the effect of dietary macronutrients on post-prandial glucose control, it appears that, to improve the calculation of the insulin bolus, the CC should be integrated with the counts of fats and proteins. Research into suitable and usable algorithms is necessary. Overall, CC may have a positive effect on metabolic control, reduce hypoglycaemia events, improve quality of life, and not influence BMI. However, more high-quality clinical trials are needed to confirm this positive impact.

## Figures and Tables

**Table 1:** Characteristics of uncontrolled studies with carbohydrate counting (CC) used in children and adolescents with type 1 diabetes (T1D).

Authors and Year	Design	Follow-Up (Months)	Sample Size	Insulin Regimen	Insulin Type (Short-Acting/Long-Acting)	Age (Years)	T1D Duration (Years)	HbA_1c_ at Baseline (mmol/mol)	HbA_1c_ at Baseline (%)	Psychosocial Measures	Weight (kg)/BMI (kg/m^2^)/BMI SDS at Baseline	Hypoglycaemia Reporting
Uncontrolled Trials												
Abaci et al., 2009 [35]	Before/after	12	9	MDI	Analogue/NPH	15	4	78	9.3	Not reported	—/—/1.1	Yes
Alemzadeh et al., 2003 [36]	Before/after	12	44	MDI	Analogue/NPH or analogue	11	5	78	9.3	Not reported	—/19.3/—	Yes
Alemzadeh et al., 2005 [37]	Before/after	12	35	MDI	Analogue/analogue	5	3	73	8.8	Not reported	—/17.1/—	Yes
Hayes et al., 2012 [38]	Before/after	9	28	MDI	Human or analogue/analogue	14 (median)	4 (median)	61 (median)	7.7 (median)	DQOL-Y	—/—/0.99	Not reported

BMI: body mass index; BMI SDS: body mass index standard deviation score; DQOL-Y: Diabetes Quality of Life for Youth scale; HbA1c: glycosylated haemoglobin concentration; MDI: multiple dose injections; NPH: neutral protamine Hagedorn.

**Table 2:** Characteristics of controlled, randomised study with carbohydrate counting (CC) used in children and adolescents with type 1 diabetes (T1D).

Author and Year	Country	Population	No. of Patients	Intervention	Control	HbA_1c_ (%) (M ± SD) Intervention/Control	Hypoglycaemia (M ± SD)	Insulin Dose (U/kg) (M ± SD)	BMI (M ± SD)	Follow Up
Enander et al. [39]	Sweden	Children and young people	45; 26/30; 14/15	Dietary education in carbohydrate counting	Dietary education in the traditional methodology (the plate exchange method)	7.43 ± 0.83 to 7.69 ± 1.00 7.70 ± 1.00 to 8.00 ± 1.00	-	0.78 ± 0.24 to 0.80 ± 0.19 0.81 ± 0.22 to 0.83 ± 0.22	-	12 months

BMI: body mass index; HbA1c: glycosylated haemoglobin concentration.
